# Supplementary figures and images for: Using deep neural networks to evaluate object vision tasks in rats
Source: PLoS Comput Biol. 2021 Mar 2;17(3):e1008714. doi: 10.1371/journal.pcbi.1008714 (PMC7954349; doi:10.1371/journal.pcbi.1008714)

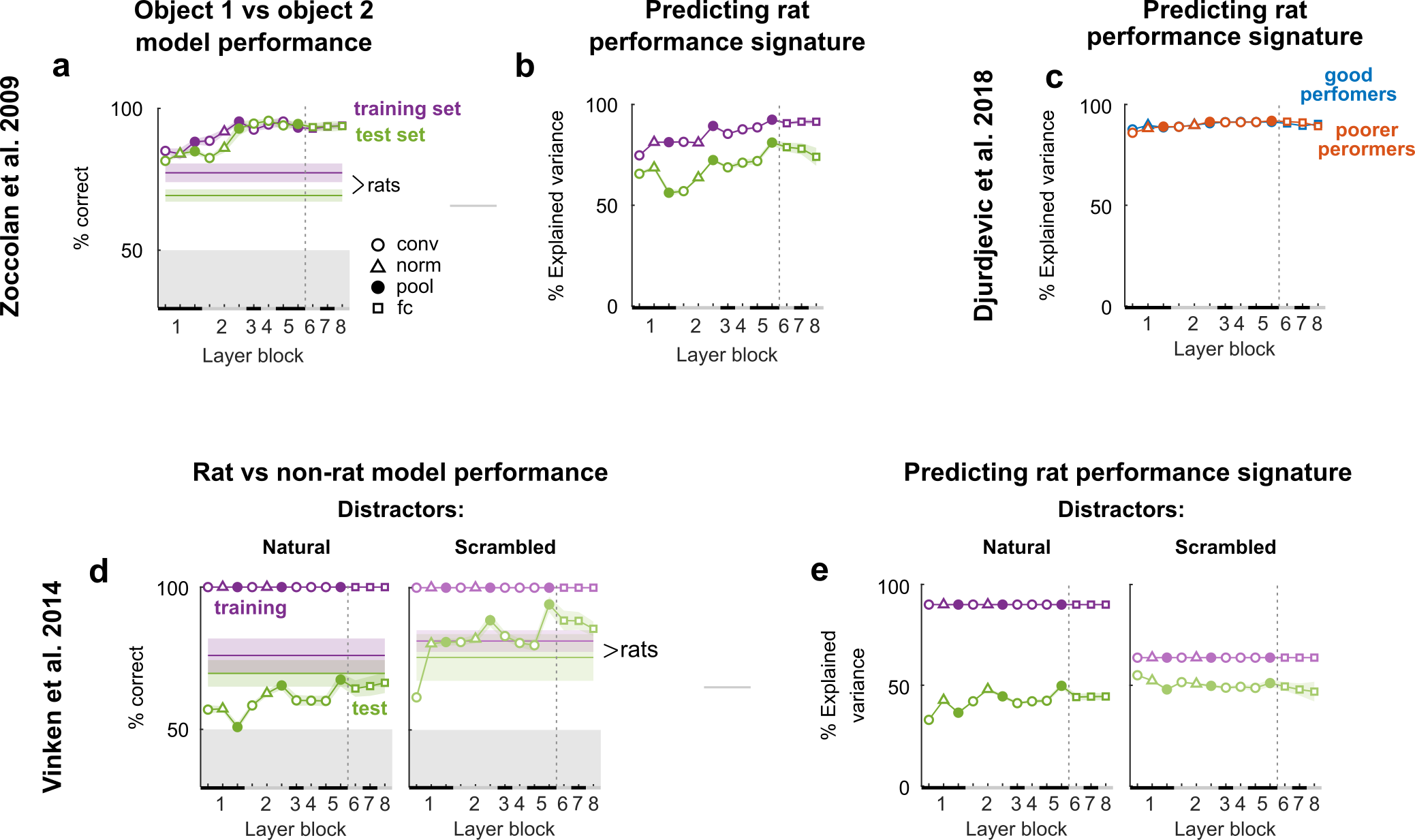

Supplement: S1 Fig — We repeated the analyses of the main figures with 10 randomly initialized AlexNet architectures (using the same scheme as the trained version, i.e., uniform Glorot initialization for the weights [56] and zero bias). (a,b) the analyses of Fig 2C and 2E. (c) the analysis of Fig 3D. (d,e) the analyses of Fig 4C–4F. All error bounds are 95% confidence intervals calculated using Jackknife standard error estimates (resampling the 10 random initializations). All other conventions match those of the corresponding figures in the main text. (TIF) [file pcbi.1008714.s001.tif]

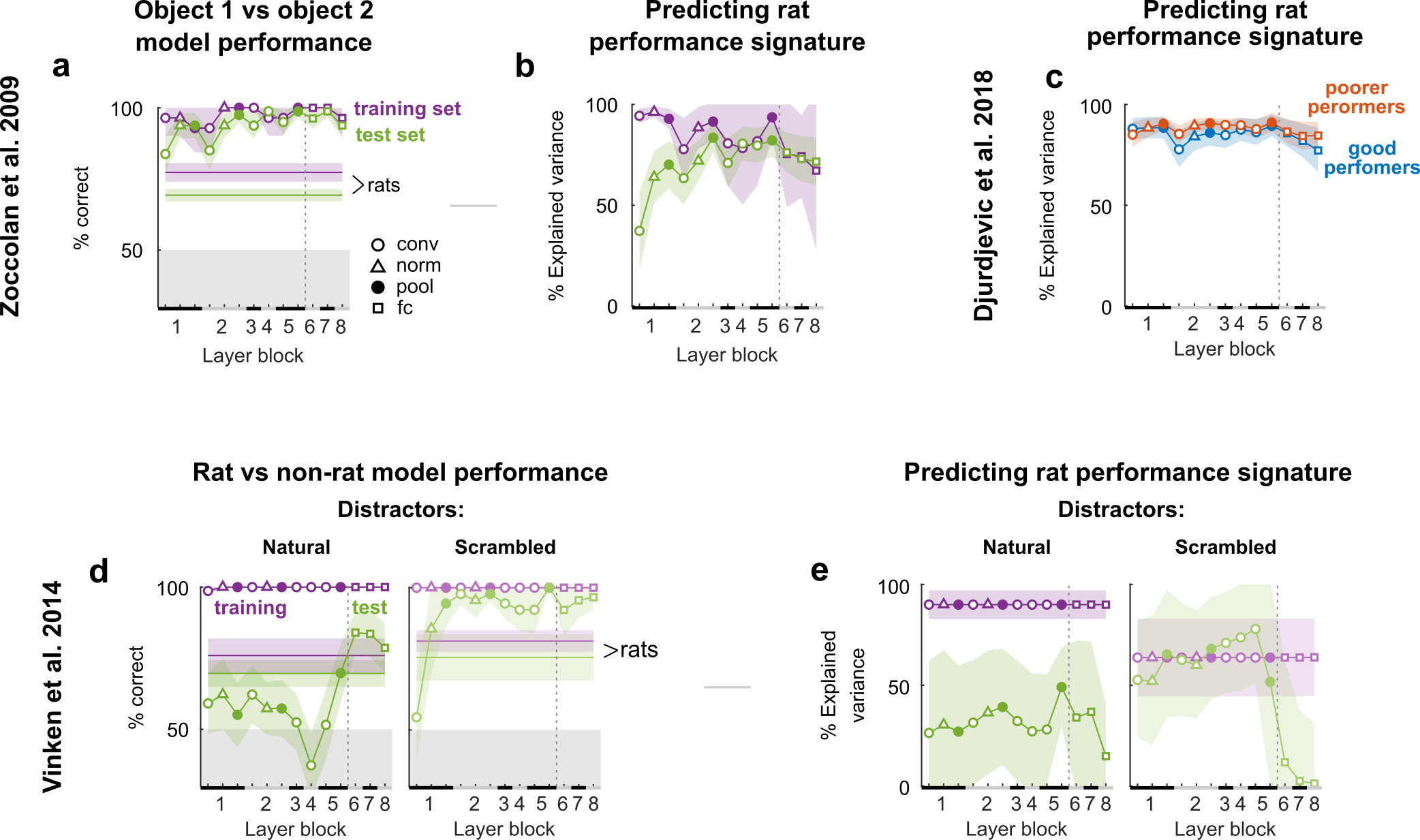

Supplement: S2 Fig — The V1 RF sizes reported in pigmented rats cover a broad range between 3 and 20+ degrees of visual angle [8,57,58]. To match DNN RF sizes in early layers with the range observed in rat V1, we downsized the stimuli so that the RF sizes in conv1 (11x11 pixels), pool1 (19x19 pixels), and conv2 (51x51 pixels) corresponded to 5, 8.6, and 23.2 degrees of visual angle, respectively, and repeated the analyses of the main figures in AlexNet. All stimuli were downsized from a default size of 227x227 pixels to match the reported presentation size in degrees of visual angle (followed by padding to the DNN input size of 227x227 pixels). (a,b) the analyses of Fig 2C and 2E, after downsizing the images to 129x129 pixels (57%) to match the largest object size of 40 degrees of visual angle. (c) the analysis of Fig 3B, after downsizing the images to 99x99 pixels (44%) to match the largest object size of 35 degrees of visual angle. (d,e) the analyses of Fig 4C–4F, after downsizing the videos to 53x53 pixels (23%) to match 24 degrees of visual angle. All conventions match those of the corresponding figures in the main text. The effects of downsizing the stimuli are most notable in (d) and (e), where the stimuli were reduced to a much lower resolution, leading to the model requiring a higher DNN layer to reach rat-level accuracies and explain most stimulus-level variance. (TIF) [file pcbi.1008714.s002.tif]

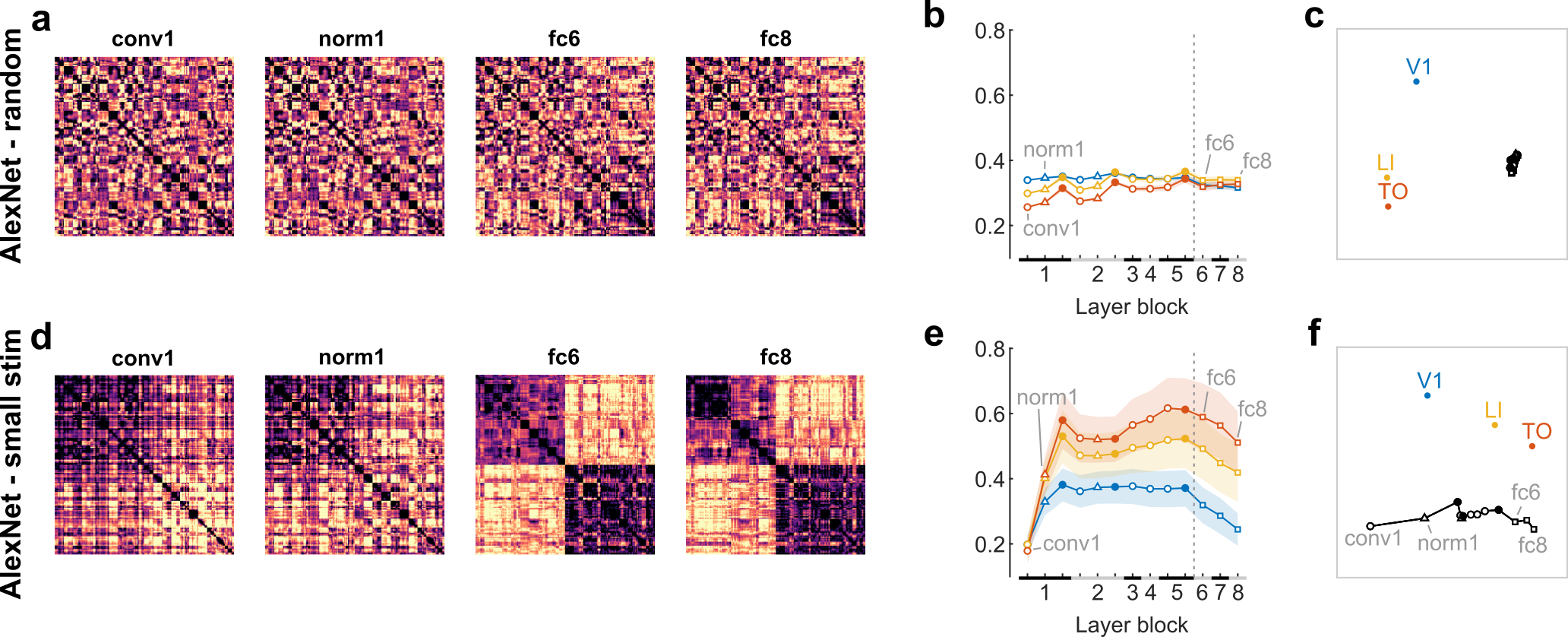

Supplement: S3 Fig — (a-c) We repeated the analyses of Fig 5D, 5G and 5J with 10 randomly initialized AlexNet architectures (see S1 Fig). Error bounds are 95% confidence intervals calculated using Jackknife standard error estimates (resampling the 10 random initializations). (d-f) The analyses of Fig 5D, 5G and 5J, after downsizing the videos to 137x137 pixels (60%) to match 62 degrees of visual angle (see explanation S2 Fig). Error bounds are 95% confidence intervals calculated using Jackknife standard error estimates (resampling neural units). All other conventions match those of the corresponding figures in the main text. (TIF) [file pcbi.1008714.s003.tif]
