# Supplementary material for: Using deep neural networks to evaluate object vision tasks in rats
Source: PLoS Comput Biol. 2021 Mar 2;17(3):e1008714. doi: 10.1371/journal.pcbi.1008714 (PMC7954349; doi:10.1371/journal.pcbi.1008714)
Supplement: Attachment — Submitted filename: PCOMPBIOL-D-20-01051.pdf [file pcbi.1008714.s004.pdf]

PCOMPBIOL-D-20-01051

Summary: In this manuscript, the authors compare the performance of deep convolutional neural networks (DNNs) with previously reported performance of rats at three visual object recognition tasks. The first task involves discriminating two objects based on their shape, under transformations of size and rotation. The second task involves identifying an object from distractors under variations in the size of the target and the distractors. The third task involved discriminating between natural movies containing rats with movies with no rats. They have used standard implementations of deep convolutional neural networks (AlexNet, VGG16, VGG11-C3D) to evaluate the performance of DNNs at these tasks. They show that the DNNs perform better than the rats at these tasks and that tasks could be performed by the first few layers (just a single layer for tasks 1 and 2) of the DNNs. They also show that the activity of these early DNN layers (as measured by the dissimilarity matrices) matches with the previously recorded neural response of rat visual areas under the third task. Based on these findings, they conclude that rodent object vision requires low level image features captured by the early stages of DNNs.

The manuscript lies within the scope of the journal. The novelty of the work is the comparison of these DNNs to rat performance at these tasks. The results are well supported. The authors need to emphasize the significance of their work and provide a better description of the underlying methods. I have the following suggestions:

1. The title claims to “evaluate the complexity of the rodent object vision”. But a mathematical definition of complexity has not been provided. It is unclear what the authors mean by complexity and how they have “evaluated” it. Further, it is also unclear whether the underlying complexity arises due to the nature of the task or the structure of the rodent visual system.

   The work compares the performance of DNNs to rats for some specific discriminations tasks, and a title along those lines would provide a better description of the work.

2. Since the number of images that are being categorized is small (compared to the size of datasets that are generally used to train DNNs) and since the authors are interested in the representation of visual information by the rodent visual system, they should try a comparison with other machine learning methods that are more interpretable in terms of the features extracted by the method. This might provide a better understanding of the representation of visual stimuli in the rodent visual pathway and provide insights for future experiments.

3. The authors should provide computer programs and the necessary data to reproduce the results. Their implementation, with linear readouts at every layer of DNN, could be a useful tool for other researchers.

4. More details in Section 2.3 (line 179-190), Section 2.4, Section 4.2.2, and Section 4.3.4 would make the manuscript more accessible to the reader (see comments below).

Minor Points:

Section 2.1 Figure 1e,f, Line 132-134, Line 469-471: Since a significant part of the results are about percent variance explained, a detailed explanation of the estimation would be helpful to the reader.

Section 2.2: How was the choice of “good performers” and “poor performers” made for the DNNs? Shouldn’t these be the same?

Section 2.3: The authors should provide more details on the computational methods.

What was the length of the video used in the training of the DNNs?

Were AlexNet and VGG16 trained on individual frames or 16 frames at a time?

Line 185-186: *“For AlexNet … 16-frame bin.”*

The authors should elaborate how they averaged outputs across the frames?

Section 2.3 Line 186-187: *“All three networks performed very similarly”*
Since the output was averaged for AlexNet and VGG16, while C3D also had temporal information, could authors comment on the temporal information in these videos/tasks?

Figure 5 (j-l): The authors should comment on the distance between V1, LI, TO and the DNN layers. Have they compared the similarity between the DNNs?

Line 280-291: *“While our results show that rodent object vision can be explained by representations in …”*

Do the results explain rodent object vision or task complexity? DNNs seem to outperform rats in all these tasks. It is unclear whether the DNN capture the behavior.

Section 4.1.2: *“We extracted the data and stimuli from the manuscripts of [1] and [5]”*
Was any attempt made to obtain the raw images and data from the authors of these publications? Would it have made any difference?

Line 442: typo “lineary”. Should it be “linearly”?

References: The formatting is not consistent.
