# Supplementary material for: Using deep neural networks to evaluate object vision tasks in rats
Source: PLoS Comput Biol. 2021 Mar 2;17(3):e1008714. doi: 10.1371/journal.pcbi.1008714 (PMC7954349; doi:10.1371/journal.pcbi.1008714)
Supplement: Attachment — Submitted filename: response_to_reviews.pdf [file pcbi.1008714.s005.pdf]

Legend:

- blue: responses to the review comments
- *green italic*: changes in the text that we quote from the manuscript
- **red bold**: line numbers in manuscript file

Reviewer #1

Summary

The paper presents computational modelling of the tasks performed in three high-profile rodent object vision studies from the existing literature. For each, the authors assess how well the rodents' behavioural task can be performed on the basis of the features at different layers of an object-categorisation-trained feedforward deep convolutional neural network (DCNN).

The text is clearly written, with a cogently explained motivation, well presented figures, and clearly explained methods and results. The work makes clever use of DCNNs to move beyond intuitions about whether a certain behaviour could be performed on the basis of, for example, V1 representations, and instead turn the question into an empirical one: what image-computable set of features can support a linear readout that would enable the behaviours shown by experimental animals? The use of real stimuli and tasks from a range of actual prior high-profile work on object vision in rodents is elegant. The findings strike an important note of caution that apparently "complex" tasks may often be performable on the basis of apparently "simple" features; computationally testing the features required to perform a task is essential. I found the paper to be generally excellent!

RE: We thank the reviewer for her/his positive evaluation.

Main comments

My only major question is about the motivation and interpretation of the modelling results: Are classification-pretrained DCNNs sound models against which to evaluate the "complexity" of a visual system?

RE: This is a highly relevant question, not only for the current paper, but also for the use of DNNs as models for biological vision in general. The short answer is that, despite their shortcomings, ImageNet-trained convolutional neural networks are our current best and most validated models of different levels of ventral stream processing. Therefore, at the moment these models are the best way to assess whether an object recognition task can be solved by lower level features, or whether more abstract representations are likely required. Once better models become well-established, of course they would be the first choice (much like

ImageNet-trained DNNs have superseded HMAX). We appreciate that this is an important nuance, and made several changes to convey this in the revised manuscript, starting with the following disclaimer in the introduction (lines **96-99**):

> "*It is important to note that the current DNN models do not capture all aspects of object vision in primates (for example see [23,24]), yet they capture ventral stream processing better than any currently available model type [25].*"

The paper is pitched as a test not of how well particular DCNNs perform as models of rodent vision, but of how "complex" rodent vision is, as indexed by how well aspects of it can be predicted from particular DCNNs. For example, the abstract proposes to test whether "invariant object recognition occurs in rodents", and Figure 1 proposes that the "lowest-level mode [layer] to solve the task indicates task difficulty". The complexity of rodent vision is certainly an interesting question, and I do agree that DCNNs can shed some light on it, in the sense that they are hierarchical models which begin as pixel representations, then move through nonlinear transformations towards object categorisations. If very early layers can well predict rodent brain and behavioural data, this suggests that there is no need to suppose sophisticated invariant object representations in the rodent brain. However, the match or non-match of later layers is harder to interpret, since it depends heavily on the training objective of the network.

RE: The match or non-match of a certain later layer is indeed hard to interpret in absolute terms. However, our approach does allow for evaluating tasks in terms of how deep we need to go in the network to find a representation where categories are well enough separated to explain generalisation in the task. There is a need for such a formalized framework to compare object recognition tasks, as previously investigators have relied on their own intuition based on untested assumptions, which the current results suggest may have been a mistake.

The current wording of the paper presents DCNNs as simply moving from "less complex" to "more complex and primate-like" representations, which is an oversimplification. The specific networks used here are pretrained on taks and datasets that have little relation to the natural tasks of either rodent or primate vision (2 models are trained to classify the main object in an image as being one of 1000 object classes, of which around 120 are different dog breeds; the other model is trained to classify sports videos into 487 sports). We know that (feedforward, supervised) DCNNs fail to capture a lot of the "visual intelligence" of primate vision (e.g. Geirhos et al (2020) https://arxiv.org/abs/2004.07780). Despite them being the best currently available models of primate vision, DCNNs fail to be "primate ventral stream like" (implication around lines 100 and 295) in lots of important ways: they are trained on ecologically unrealistic objectives that only capture a tiny portion of what vision does, and perhaps as a result are more fragile, susceptible to noise, reliant on texture cues, etc, than primate vision.

RE: This is again a very good point. We never intended to imply that the models we use successfully capture all aspects of primate object vision. To make that make that point clear, we changed the wording on lines **141-145**:

> "As the sequence of layers contains increasingly abstract representations that are increasingly more *useful* for invariant visual object recognition, each successive model simulates a visual system which has access to increasingly higher level *representations that each map best onto successive stages of the* primate ventral stream *[14,15,30]*."

And on lines **468-472**:

> "Regardless, DNNs are mechanistic models that can capture the steps of information processing required to solve visual recognition *of the main object in natural images. While these steps of information processing in DNNs do not capture all aspects of primate vision [23,24],* on a macroscale *they* do map onto successive stages of primate ventral stream processing *[14,15,30]*."

In addition, we performed a new analysis which confirmed that the separability of the stimulus classes used in the behavioral experiments indeed increased as a function of layer number. While this does not prove anything about how accurate ImageNet-trained DNNs are as models of primate vision, it does confirm that also for the stimuli used in the present study these networks behave like one would expect from an invariant visual object recognition system (new panel Fig 1c and lines **145-147**):

> "*Indeed, for the stimulus sets considered in the present study we found that higher DNN layers showed increasingly better separation for object identity and category (Fig 1c), even though these models were trained on ImageNet [31].*"

More fundamentally, models of this kind are classifiers, moving from pixels to whatever carving-up of category space they have been trained to perform. The extent to which this leads to the development of "complex" representations (e.g. view invariance) depends more on the training data and task than on the type of model. Although the 1000-class Imagenet task does lead to networks with decent position and view invariance, it also produces networks which over-rely on textures (Geirhos et al (2018) https://arxiv.org/abs/1811.12231), likely because it can be reasonably well solved on the basis of local image fragments (Brendel & Bethge (2019) https://arxiv.org/abs/1904.00760). Models trained on different tasks will learn different image transformations. It is therefore inaccurate to view the layer of a particular DCNN as a straightforward proxy for "complexity" or "difficulty" of a visual task.

RE: We do feel that the phrasing "complexity of rodent vision" (in particular in the title) may have wrongly given the impression that our results lead to clear conclusions about the nature of the

representations found in the rat visual cortex. To avoid any such misunderstandings, we removed this phrasing from the text and changed the title to the more appropriate:

> "*Using deep neural network models to evaluate object vision tasks in rats.*"

In addition, we added supplementary figures (Fig S1 and S3) showing the results for a randomly initialized DNN, which provides a baseline to assess how training on an ImageNet classification task affects the results (see also our response to Reviewer #3).

All of which is to say: object-classification supervised DCNNs are obviously the best models of high-level vision we currently have, both in terms of their sheer accuracy and their match to primate brain and behavioural data. However, in using them as a yardstick for the "complexity" or "invariance" of another model visual system, it's important to acknowledge that the features in the models used here arose from training on very limited tasks, which likely don't align with the goals of a rodent. It is important to specify that the DCNNs considered here are *supervised feedforward 1000-class object-categorisation networks* - a very different "level of abstraction" relationship would likely hold across the layers in e.g. a generative adversarial network or a variational autoencoder. It would be very interesting to see how an unsupervised DCNN model fared, but I think this point can be adequately covered by changes to the text in the Abstract, Introduction, Figure 1, and perhaps Discussion, without new experiments.

RE: We made the following changes in the discussion to explicitly acknowledge this point (lines **458-466**):

> "It is important to note that the DNNs used in this study *are supervised, feedforward neural networks trained on object recognition, which may constrain the features that are encoded by each layer. These models were* not designed to model rodent vision *[although the overall conclusions did hold when the stimuli were resized so that receptive field sizes in early DNN layers match V1 receptive field sizes (**Fig S 2** and **Fig S 3**d-f)], because they are neither optimized for the same goals, nor are they* subject to the same biological constraints of either the rodent or primate visual systems. Therefore, we do not claim that *feedforward* DNNs trained on object recognition are optimal model*s* of the putative rodent ventral stream, which potentially encodes different information compared to the primate ventral stream."

Minor comments

- If features are extracted from each layer pre-ReLU, does this mean that the conv1 layer is simply a linear combination of pixels? It might be helpful to see a raw pixel model as a baseline (i.e. linear classifier is trained to perform task directly on pixels of image), since performance is so high for all layers in many plots. e.g. line 122 "we...found that this level of generalisation

required surprisingly little processing" - does it require *any* processing, or is this a task that can be trivially solved with no nonlinear image transformations at all?

RE: Yes, the fact that we extract features before the ReLU indeed implies that a linear operation (input -> convolution -> pca -> classifier) is sufficient to solve and generalize the task. A raw pixel model would be another model without nonlinearities, which only lacks the trained convolution layer (input -> pca -> classifier). We did this for the two behavioral experiments with separate training sets and found results that are generally consistent with earlier layers:

> "*Even a model trained on a pixel representation achieved 93% correct on the training set and 89% on the test set.*" (lines **189-190**)

> "*A model trained on a pixel representation achieved only 60% for scrambled distractors and 66% for natural distractors on the test set.*" (lines **312-314**)

- In Figure 3, the faint curved lines connecting the network layer labels in 3b to the y-axis titles of 3d look distractingly like confidence bounds on the data plotted in 3b, and took me a while to interpret. May be better removed altogether?

RE: That is a good point. We have removed these arrows to avoid any potential confusion.

- Around line 285 when interpreting how the layerwise correspondence with rat neural data relates to that found with human/non-human primate brain data, it seems as if most human brain data now point to something fairly similar to the correspondences shown for rats in Figure 5. E.g. peak correspondences are found for mid-to-late layers, falling in the latest layers, in posterior VTC (https://www.nature.com/articles/s41598-020-59175-0), LOT and VOT for several stimulus sets (https://doi.org/10.1101/2020.03.12.989376) and a broad IT swath (https://doi.org/10.1101/2020.05.07.082743).

RE: These references indeed add to the evidence that often also in humans representations in a large part of higher visual cortex are best captured by late convolutional layers. It appears like whether or not this correspondence peaks at fully connected layers depends on several factors, including the stimulus set, where in IT the data were recorded, and the network architecture. We now include this nuance in our discussion together with the suggested references, on lines **449-453**:

> "For the similarity between neural representations in primate inferotemporal cortex and DNN layers the story is less consistent: in some cases it does peak at the fully connected layers [21,*41*], whereas in other studies it peaks at the last convolutional layer [15,17,42,43], suggesting that it might depend on the particular stimulus set*, how anterior in the ventral stream the neural data were recorded [21], and/or the network architecture [44].*"

Typos / wording

- line 350 / 352: "at one hand"? Not "on one hand"?

RE: We have corrected this error.

- line 358: "rats WERE trained"

RE: We have corrected this error.

Reviewer #2

Summary

In this manuscript, the authors compare the performance of deep convolutional neural networks (DNNs) with previously reported performance of rats at three visual object recognition tasks. The first task involves discriminating two objects based on their shape, under transformations of size and rotation. The second task involves identifying an object from distractors under variations in the size of the target and the distractors. The third task involved discriminating between natural movies containing rats with movies with no rats. They have used standard implementations of deep convolutional neural networks (AlexNet, VGG16, VGG11- C3D) to evaluate the performance of DNNs at these tasks. They show that the DNNs perform better than the rats at these tasks and that tasks could be performed by the first few layers (just a single layer for tasks 1 and 2) of the DNNs. They also show that the activity of these early DNN layers (as measured by the dissimilarity matrices) matches with the previously recorded neural response of rat visual areas under the third task. Based on these findings, they conclude that rodent object vision requires low level image features captured by the early stages of DNNs.

The manuscript lies within the scope of the journal. The novelty of the work is the comparison of these DNNs to rat performance at these tasks. The results are well supported. The authors need to emphasize the significance of their work and provide a better description of the underlying methods. I have the following suggestions:

1. The title claims to "evaluate the complexity of the rodent object vision". But a mathematical definition of complexity has not been provided. It is unclear what the authors mean by complexity and how they have "evaluated" it. Further, it is also unclear whether the underlying complexity arises due to the nature of the task or the structure of the rodent visual system. The work compares the performance of DNNs to rats for some specific discriminations tasks, and a title along those lines would provide a better description of the work.

RE: We agree that it's better to avoid using "complexity" like this without formal definition, so we removed the ambiguous use of this word from the text and also changed the title to the more appropriate:

> "*Using deep neural network models to evaluate object vision tasks in rats.*"

This title more accurately reflects the goal of using DNNs to formally compare behavioral results and get a better grasp on whether these data actually provide support for high level, highly invariant representations or not.

Regarding the question of "whether the underlying complexity arises due to the nature of the task or the structure of the rodent visual system", we do think the evidence is constrained by the

task difficulty. We briefly discussed this already but made this point more clear with the following changes (lines **498-506**):

> "Finally, it is important to emphasize that the fact that we have not found any evidence for truly high-level visual object recognition behavior does not imply that it is not there or cannot be there. *For example, a high generalization performance in the video categorization experiment required higher layer representations than the other experiments, underlining that the evidence we have is not only limited by the capabilities of the rat visual system, but also by the nature of the task.* It is possible that none of the studies discussed here really pushed the limits of rodent object vision. A road ahead for addressing this question is to use DNNs to construct stimulus sets and design paradigms that explore the boundaries of rodent vision by getting the best out of them."

2. Since the number of images that are being categorized is small (compared to the size of datasets that are generally used to train DNNs) and since the authors are interested in the representation of visual information by the rodent visual system, they should try a comparison with other machine learning methods that are more interpretable in terms of the features extracted by the method. This might provide a better understanding of the representation of visual stimuli in the rodent visual pathway and provide insights for future experiments.

RE: The small number of stimuli is unfortunately a limitation of the behavioral experiments that the current study is based on: 2 objects under various transformations in case of Zoccolan et al. 2009; 12 objects at different sizes in case of Djurdjevic et al. 2018; or 55 videos (20 of rats, 20 of various objects, and 15 scrambled) in case of Vinken et al. 2014.

Despite the low number, the stimuli themselves are relatively complex to very rich (in case of the videos) in terms of visual features. We're not sure which more interpretable machine learning methods the reviewer had in mind, but we expect that comparing the performance of a battery of interpretable features could lead to a substantial number of false positives. That is, for such small stimulus sets, it would not be too hard to find even random features that generalize from the training to test set by chance.

3. The authors should provide computer programs and the necessary data to reproduce the results. Their implementation, with linear readouts at every layer of DNN, could be a useful tool for other researchers.

RE: We will include code for an implementation of the model on the paper's OSF page (at https://osf.io/4w39d/) and will be adding all of our data used to draw the conclusions, as required by the journal's guidelines. We added references to this link on lines **558** and **645**.

4. More details in Section 2.3 (line 179-190), Section 2.4, Section 4.2.2, and Section 4.3.4 would make the manuscript more accessible to the reader (see comments below).

RE: We made several changes to address this criticism, as explained in detail in the "Minor points" below. In addition, we made the following clarifications about the RDM calculation in Section 4.2.2 (lines **571-579**):

> "In short, *for each of the 20 five second videos we considered the first nine 16-frame bins (533ms each – together covering 144 out of the full length of 150 video frames, or 4.8 out of 5 seconds) to match the temporal bins explained under Computational modeling. This yielded a total of 180 16-frame bins. Next, for each of these* 16-frame video bins*, we calculated a neural response vector with the average standardized firing rate of each* single and multi-unit *response, taking into account* a temporal shift corresponding to the response latency which was estimated separately for each neuron or site [see 33]. This resulted in *180* response vectors *(one* per 16-frame bin *of each* stimulus*)*, which were correlated pairs-wise (Pearson r) in order to obtain RDMs with distances 1−r."

Minor points

Section 2.1 Figure 1e,f, Line 132-134, Line 469-471: Since a significant part of the results are about percent variance explained, a detailed explanation of the estimation would be helpful to the reader.

RE: This information was in the methods Section 4.3.4 "Predicting behavioral performance signatures" (previously "patterns"). We added an equation and a clearer definition of "behavioral performance signature" in that section (lines **653-667**):

> "*The probability of correct response by the rats was not the same for every trial, but varied depending on the presented stimulus (i.e. size and rotation in [1], object and size in [5], and category exemplar in [6]). We call this pattern of percentages correct across training or test images in the task the behavioral performance signature [26].* We used a linear regression approach to assess whether a linear mapping could predict *these* behavioral performance *signatures* across stimuli from activation patterns in DNN layers. […] The accuracy of each model at predicting behavioral performance *signatures* was assessed by calculating the percentage explained variance as the squared Pearson correlation between predicted *performance values* y^{pred} and observed (logit-transformed) performance values y^{obs}: $corr(y^{pred}, y^{obs})^2$."

We also added a clear definition of "behavioral performance signatures" in Section 2.1 for clarity (lines **193-201**):

> "*The performance of the rats was not the same for all combinations of size and azimuth-rotation: performance was highest for the combination of the most common viewpoint and size in the training set (i.e. the center object in the purple "cross" in Fig*

2a,b), and decreased for objects that deviated from that combination. We call this pattern of performances across object transformations (or – more generally – across images) the behavioral performance signature. To assess whether DNN representations could capture the behavioral results beyond overall generalization accuracy, we tested whether a linear combination of the activation patterns in a DNN layer could also accurately predict *this* behavioral performance *signature* (Materials and Methods, Computational modeling, Predicting behavioral performance *signatures*).”

Section 2.2: How was the choice of “good performers” and “poor performers” made for the DNNs? Shouldn’t these be the same?

RE: This division was part of the original paper (as specified in the first paragraph of Section 2.2), and we fitted a linear mapping separately for good and poorer performers. We made the following changes to the text to clarify this point (lines **221-249**):

“The authors found that there were "good performers", which performed above chance for *all* distractors *at a size of 30°*, and "poorer performers", which performed below chance for *more* challenging distractors *(e.g., the T-shape highlighted in blue in **Fig 3**a)*. [...] We calculated how much of the object and size-level variance in behavioral performance could be explained by the DNN layer activations*, by fitting a linear mapping separately for the data from good and poorer performers (*using the data that was displayed in Figure 1 and 2 of Djurdjevic et al. [5]*; Materials and Methods, Computational modeling, Predicting behavioral performance signatures)*.”

Section 2.3: The authors should provide more details on the computational methods. What was the length of the video used in the training of the DNNs? Were AlexNet and VGG16 trained on individual frames or 16 frames at a time? Line 185-186: “For AlexNet … 16-frame bin.” The authors should elaborate how they averaged outputs across the frames?

RE: The length of each full video was 5s or 150 frames (of which we divided the first 144 in nine 16-frame bins). For consistency with VGG11 - C3D, AlexNet and VGG16 based models were trained to classify the 16-frame averaged bins. To get these averaged bins, we took the mean output of each unit across the 16 frames of a bin. We added this information in Section 2.3 (lines **304-308**):

“*For training the DNN-based models, we took the first 144 frames of the total of 150 frames (4.8 out of 5s) of each video and split those into nine 16-frame bins. The inputs to each classifier were the time-averaged activations of each 16-frame video clip (Materials and Methods, Computational modeling, Feature extraction).*”

In addition, we expanded the methods Section 4.3.2 “Feature extraction” as follows (lines **607-612**):

> “In *the* case of videos, activations were averaged over time within each 16-frame bin *before dimensionality reduction using principal component analysis. For VGG11-C3D this was done by taking the mean activation of each spatiotemporal convolutional kernel across the temporal dimension. For AlexNet and VGG16 (which were pre-trained on static images) we averaged outputs across frames for each 16-frame bin, by taking the mean activation of each unit across frames.*”

Section 2.3 Line 186-187: “All three networks performed very similarly” Since the output was averaged for AlexNet and VGG16, while C3D also had temporal information, could authors comment on the temporal information in these videos/tasks?

RE: That is a good point. Even though for VGG11-C3D we average filter activations across the temporal dimension, each convolutional filter combines weighted information across increasingly larger time windows. The fact that image trained DNNs do not perform any worse on this task suggests that in principle such temporal information is not necessary to perform the task and successfully generalize. We discuss this briefly in the revised Section 2.3 (lines **300-309**):

> “VGG11-C3D is a convolutional neural network with 3D spatio-temporal filters (16-frame temporal bins) and thus able to also encode temporal features [32]. *The features encoded by VGG11-C3D range from moving edges/blobs and changes in orientation or color, to more complex motion patterns such as moving circular objects or biking-like motions [32]. For training the DNN-based models, we took the first 144 frames of the total of 150 frames (4.8 out of 5s) of each video and split those into nine 16-frame bins. The inputs to each classifier were the time-averaged activations of each 16-frame video clip (Materials and Methods, Computational modeling, Feature extraction).* All three networks performed very similarly*, suggesting there was no real advantage of the motion features encoded by VGG11-C3D for this task.*”

Figure 5 (j-l): The authors should comment on the distance between V1, LI, TO and the DNN layers. Have they compared the similarity between the DNNs?

RE: We included a comment on this in Section 2.4 (lines **396-397**):

> “*On average, the distance between the neural and DNN layer RDMs was 2.27 times the average distance between DNN layer RDMs from the three different DNN models.*”

Line 280-291: “While our results show that rodent object vision can be explained by representations in …”

Do the results explain rodent object vision or task complexity? DNNs seem to outperform rats in all these tasks. It is unclear whether the DNN capture the behavior.

RE: We made the following changes to be more explicit (line **445-446**):

> "While our results show that *generalization in* rodent object vision *experiments* can be explained by representations in…"

Section 4.1.2: "We extracted the data and stimuli from the manuscripts of [1] and [5]" Was any attempt made to obtain the raw images and data from the authors of these publications? Would it have made any difference?

RE: Because we were able to extract all the critical information (up to an arbitrary precision) from the manuscript, we did not end up asking the authors for the raw images/data and we do not think it would have made any difference with respect to the conclusions. We did send a draft to Davide Zoccolan, who is an author on both the behavioral studies which were not from our lab. Davide provided helpful comments on this work, which we have acknowledged in the "Acknowledgements" section of the paper.

Line 442: typo "lineary". Should it be "linearly"?

RE: Yes, we corrected this typo.

References: The formatting is not consistent.

RE: We have properly formatted the references in Vancouver style.

Reviewer #3

Summary

The manuscript "Using deep neural networks to evaluate the complexity of rodent object vision" by predicting behavioral data of rats from three different paradigms from convolutional layers of three different DNNs, and by using a representational dissimilarity analysis between DNN at different layers and rat neurophysiological data from different areas. The authors conclude that rodent vision can be captured by mid-level layers of DNNs, earlier layers than required for primate vision, and thus requires lower complexity.

Major comments

I have to admit I have some trouble with this paper.

First of all, it could improve on clarity. It's really hard to understand the exact stimulus paradigms from the paper. Yes, they can be found in the orignal submissions, but it would be good to summarize each paradigm in a plot to get the reader on board and not force her/him to look up technical details in other papers.

RE: Thank you for the useful feedback. Below we describe how we have added the information that was missing or unclear.

The behavioral paradigms are explained under Section 4.1.1 “Task paradigms”. We now made sure we refer to this methods section at each subsection of the results:

> “In this study, rats were first trained to discriminate *between* two different objects and then to tolerate variations in size *(15 to 40 degrees of visual angle)* and azimuth-rotation *(60° left to 60° right), using a yes-no task in an operant box with liquid rewards (see Materials and Methods, Behavior, Task paradigms).* At each trial, one object was presented and the rat had to indicate the object identity by licking either a left or right feeding tube.” (Section 2.1, lines **159-163**)

> “In this study, rats were trained to discriminate a reference object from 11 distractor objects at different sizes *ranging from 15 to 35 degrees of visual angle* (**Fig 3**a)*, using a yes-no task in an operant box with liquid rewards (see Materials and Methods, Behavior, Task paradigms).*” (Section 2.2, lines **217-220**)

> “In this study, rats were trained *on a two-alternative forced choice task* in a visual water maze *(see Materials and Methods, Behavior, Task paradigms)* to classify *five-second* videos featuring a rat (target category), from phase scrambled versions of the target videos and target-matched natural distractor videos featuring various moving objects.

The videos were 24 degrees of visual angle as seen from the choice point where the maze splits in two arms [6]." (Section 2.3, lines **265-269**)

In addition, we specify the stimulus presentation details of the neural experiment in the results Section 2.4 (lines **352-357**):

"In this experiment, we presented the *10* natural videos of the training set in Fig 4a and their *10* scrambled counterparts *in randomized order* to awake, passively watching rats which were never trained with these videos. *The videos were shown at sizes ranging from 50 to 74 degrees of visual angle (as the eye-to-stimulus distance varied according to the position on the screen, which was optimized for each recording site's receptive field location) separated by a 2 s blank screen, with 10 repetitions per video.*"

Furthermore, important details seem often hidden behind unnecessary high level language and could easily be made more concise. To give an example: You talk about "behavioral patterns". That could mean anything from ethogram to single trial behavioral responses. I think in the current manuscript it's neither, but I also couldn't find out what it is exactly. Things like that make the manuscript hard to read and understand.

RE: "Behavioral performance patterns" refers to the variability in performance across stimuli. For clarity, we changed the word "pattern" to the more noteworthy term "signature", which has been used before in this context by Rajalingham et al. 2018, and added a clear definition of "behavioral performance signature" in Section 2.1, where it is first introduced (lines **193-197**):

"*The performance of the rats was not the same for all combinations of size and azimuth-rotation: performance was highest for the combination of the most common viewpoint and size in the training set (i.e. the center object in the purple "cross" in **Fig 2**a,b), and decreased for objects that deviated from that combination. We call this pattern of performances across object transformations (or – more generally – across images) the behavioral performance signature.*"

And in the methods in Section 4.3.4 "Predicting behavioral performance signatures" (lines **653-656**):

"*The probability of correct response by the rats was not the same for every trial, but varied depending on the presented stimulus (i.e. size and rotation in [1], object and size in [5], and category exemplar in [6]). We call this pattern of percentages correct across training or test images in the task the behavioral performance signature [26].*"

Finally, important details and measures are missing. For instance, I couldn't find an exact description of the behavioral data (only that it was extracted from the plots of the original

manuscripts). What are the inputs? What's exactly measured? This is important to understand what you are analyzing.

RE: The behavioral data are the percentages correct trials per stimulus type/target distractor combination. We now specify this in Section 4.1.2 “Data and stimulus extraction” (lines **550-559**):

> “The *behavioral data* were copied from the *values displayed* in Fig. 2B of Zoccolan et al. [1] (*percentage correct based on 70-90 trials for each size and azimuth-rotation and per animal, averaged across* N = 6 rats), and extracted from Figures 1C *and 1D* of Djurdjevic et al. [5] using WebPlotDigitizer 4.2 (https://apps.automeris.io/wpd/; *percentage correct based on an unspecified number of trials for each size of the target object and of three distractor objects, as well as for the 30° size of all remaining distractor objects, for each of* N =6 rats). For the analyses of the natural video categorization experiment, we used the original movies from Vinken et al. [6], and had access to the original data *which are available at https://osf.io/4w39d/ (percentage correct based on 14-49 [M = 30, SD = 10] trials for each target-distractor combination pooled across* N = 5 rats).”

In addition, almost all plots are missing any sort of error bars to judge whether the difference (e.g. between different layers) is significant or not.

RE: We added error bounds showing 95% confidence intervals calculated using Jackknife standard error estimates to all figures that previously did not have error bounds (Figs 2, 3, and 4).

The major issue that I have is that I have a hard time believing in the conclusions. If I had to rephrase what was done in the paper it would be that midly complex tasks (or rats' behaviors on it) were fitted with very powerful deep networks on likely very few training trials. Because the test error peaks at mid-level layers of these networks, it is suggested that the rat visual system is less complex than primates (because they peak later). However, there are almost no controls or alternative explanations suggested by the authors. To list a few that I would consider:

RE: We thank the reviewer for her/his suggestions, and did extensive new analyses to address the concerns raised by the reviewer, which we detail in our responses below.

- The training performance of these networks is basically flat everywhere. This means that the task is likely linearly separable on almost all layers. This means that there is likely also a linear function that can solve the test samples perfectly on almost every layer, you just cannot find it from limited training data. I am positive that if you trained on the test trials as well you would find that function. However, if that's the case, the conclusion changes, because the reason for the peak at a certain layer is not representational complexity but your ability to fit the data on limited trials.

RE: The question is not whether the two classes of stimuli used in the experiments are linearly separable – any set of N randomly labeled, non-collinear points will be linearly separable in ≥N-1 dimensional space, so this point could be made for virtually any behavioral stimulus set given the dimensionality of (early) DNN layers (and visual areas). The critical question is whether a hyperplane optimized for a given training set (i.e. the stimuli used to train rats) will generalize to a new set unseen by the classifier (i.e. the stimuli used to test generalization in rats). Thus our conclusions do not depend on the linear separability of the test set (as this is trivial), but on how well a hyperplane that was optimized for the training set generalizes to the test set.

That being said, we realize "complexity" of representations is not the right phrasing to express the goal of our work here, which is to evaluate task difficulty with DNNs trained on object recognition as a framework to better understand what evidence is contained in previous experiments. For this reason we changed the use of the word complexity in the title and throughout the paper (see also our responses to Reviewers #1 and #2).

- Predictive performance is probably not a very fine grained score to distinguish between different feature complexities in networks. For me, that's the main conclusion of the Cadena paper you discuss. If you have a complex feature space you can fit many things well, in particular if you have few trials. For that having controls like randomly initialized networks like in Cadena et al. is important. If you run those, make sure to use a batch norm after the final layer to counter possible large differences in scale in the random architecture.

RE: We agree that this is a very useful addition. We performed all analyses with randomly initialized versions of AlexNet and included the results in two new supplementary figures. The results for the behavioral experiments are shown in Fig S1, and summarized in the main text (lines **323-335**):

> "*The results show that stimulus representations of higher DNN layers are required to achieve generalization performance levels on the rat/non-rat classification task that are comparable to rats. Could this layer effect merely be a consequence of the hierarchical architecture of the DNNs, which have properties that systematically change across layers? Even without training, DNNs can provide a powerful set of features, and trends across layers in the ability to capture experimental data can be explained by the number of linear-nonlinear stages rather than the task-optimization of the network [33]. To test this, we repeated our simulations of the behavioral experiments using randomly initialized DNN models (**Fig S 1**). Random networks achieved high generalization in the object classification task of **Fig 2** and the video classification task of **Fig 4** with scrambled distractors but not with natural distractors, where performance levels stayed below those of rats even for the highest layers. Thus, the layer-wise increase in generalization performance in Fig 4c depends on the increasingly more abstract features that were learned when the network was trained on image classification.*"

The results for the neural data are shown in Fig S3a-c, and summarized in the main text (lines **400-402**):

> "*The strong interaction between DNN layer and cortical area could not be explained by randomly initialized DNNs (**Fig S 3**a-c), suggesting a significant role of the specific features that were learned when the network was trained on image classification.*"

Furthermore, you could try to use other meaures to check whether the behavior of the networks is consistent with the behavior. A useful reference might be "Geirhos, R., Meding, K., Wichmann, F. A. (2020). Beyond accuracy: quantifying trial-by-trial behaviour of CNNs and humans by measuring error consistency. arXiv preprint arXiv:2006.16736."

RE: This is why we performed our analyses on predicting variability in performance across stimuli/object transformations (i.e. the "behavioral signatures" explained in our reply to a question above) from DNN representations. The results show that in some cases later (conv) layers can account increasingly better for these behavioral performance signatures (Fig 2), in other cases the prediction accuracy peaked at middle convolutional layers (Fig 4).

- What about the confounder of task complexity? You mention it in the Discussion but it would be important to control for it.

RE: One of the main goals of this paper was to use DNNs to evaluate the behavioral object recognition tasks that rats have been able to successfully solve, in order to get a better understanding of what these animals are visually capable of. From this point of view, task complexity or task difficulty is not a confound we need to control for, but exactly that which we are interested in assessing using DNNs as a principled framework.

Task difficulty provides an upper bound to what evidence can be taken away from a behavioral experiment: if a task is trivial it cannot be taken as evidence for complex visual capabilities. More specifically, if for a given classification task a linear classifier can easily generalize from a V1-like representation, we argue that perhaps this task should not be considered as evidence for advanced visual object recognition behavior. A principled evaluation of task difficulty is a prerequisite for answering the question of whether there may be evidence for more advanced visual object recognition capabilities in rat behavioral studies or not.

We now explain this goal more clearly with the following changes in the introduction:

> "However, *the extent to which previous object recognition experiments in rats probed higher-level vision has never been tested empirically. That is, to what extent did these classification tasks actually require an abstract, invariant representation of the visual stimuli? From previous work we know that rats are experts at finding and using the lowest-level feature that is predictive of a correct response in a discrimination task [10]. Thus,* in order to appreciate the capabilities of the rodent visual system, *it is critical* to

understand the *minimum* level of abstraction that *is required to solve the tasks that these animals are able to perform*. Already from the first landmark study by *Zoccolan et al. [1]*, it has been argued that the behavior is unlikely to be based upon representations found in *the* primary visual cortex (V1). *However, the level of invariance found in the later stages of the primate ventral stream may not be necessary to explain generalization in rodent object vision experiments, as even similarly small sized Marmoset monkeys far outperformed rats on the same task [11].*" (lines **51-62**)

"*We evaluated for each DNN layer's representational space whether a linear classifier could generalize from the stimuli used in the training phase of experiments in rats, to the novel stimuli used in the test phase. We reasoned that if generalization is successful in early DNN layer representations, the task does not require high-level, invariant object representations, and thus one should be careful in interpreting generalization performance of rodents as evidence for invariant object recognition.*" (lines **104-109**)

In the discussion we further underline that the present approach does not imply that there could not be a more complex task which could reveal evidence for higher-level visual capabilities (lines **498-503**):

"Finally, it is important to emphasize that the fact that we have not found any evidence for truly high-level visual object recognition behavior does not imply that it is not there or cannot be there. *For example, a high generalization performance in the video categorization experiment required higher layer representations than the other experiments, underlining that the evidence we have is not only limited by the capabilities of the rat visual system, but also by the nature of the task.*"

- What about image scale in RDS? Do the similarity measures change with image scale? Deep networks have a strong correlation between spatial size of a feature and feature complexity. Rats likely have large receptive fields compared to primates (in mice it's definitely true; I don't know rats well enough). So this confounder needs to be controlled for by presenting different scaled input images/videos when computing the RDS.

RE: In human and monkey studies the match in receptive field (RF) sizes between the visual system and DNNs is typically not taken into account. It is true that spatial RF sizes are larger in rats compared to macaques or humans. To address this issue we redid all analyses with AlexNet after resizing the stimuli to match early DNN layer RF sizes with those reported in V1 of pigmented rats:

"*The V1 RF sizes reported in pigmented rats cover a broad range between 3 and 20+ degrees of visual angle [8,57,58]. To match DNN RF sizes in early layers with the range observed in rat V1, we downsized the stimuli so that the RF sizes in conv1 (11x11 pixels), pool1 (19x19 pixels), and conv2 (51x51 pixels) corresponded to 5, 8.6, and 23.2 degrees of visual angle, respectively, and repeated the analyses of the main figures in*

AlexNet. All stimuli were downsized from a default size of 227x227 pixels to match the reported presentation size in degrees of visual angle (followed by padding to the DNN input size of 227x227 pixels)." (legend of Fig S2, lines **857-863**)

The results for the behavioral experiments are shown in Fig S2, and for the neural data in Fig S3d-f. If anything, they suggest that when the stimuli are drastically reduced in size, later layers are required to explain the data (Fig S2d,e), but not to an extent that it changes the conclusions that we draw. We refer to these supplemental results in the discussion (lines **460-464**):

"*These models were* not designed to model rodent vision *[although the overall conclusions did hold when the stimuli were resized so that receptive field sizes in early DNN layers match V1 receptive field sizes (Fig S 2 and Fig S 3d-f)], because they are neither optimized for the same goals, nor are they* subject to the same biological constraints of either the rodent or primate visual systems."

- Finally, it's known that deep networks use different strategies to solve problems (i.e. they are texture biased. See work by Geirhos and collegues or by Brendel on BagNets). The reason for that is that even a large scale dataset like imagenet can be solved in many ways and texture seems to be easier to learn for those networks. I feel you conclusions rests on the assumption that the networks and the rats solve the task in the same way, but that's likely not the case. How does this affect your conclusion? This needs to be discussed, analyzed, and controlled for.

RE: Our main assumption is that we can reasonably use standard DNN models trained on object recognition as a proxy to evaluate object recognition task difficulty. This assumption is motivated by the fact that we know that object classes are increasingly separable in deeper layers of DNNs. We now also show that this is true for the specific stimulus sets used in the present paper (new panel (c) in Fig 1 and lines **145-147**):

"*Indeed, for the stimulus sets considered in the present study we found that higher DNN layers showed increasingly better separation for object identity and category (**Fig 1**c), even though these models were trained on ImageNet [31].*"

An equally important motivation is the extensive validation in the neuroscience literature which suggests that, despite clear shortcomings, the progression toward deeper DNN layers maps to a certain extent on stages of primate ventral stream processing, while also capturing important (though certainly not all) aspects of primate visual object recognition behavior. Thus, despite their shortcomings and room for improvement, DNN models trained on object recognition are our current best models of visual object recognition in the primate brain. We made changes to be more explicit about these shortcomings in the introduction (lines **96-99**):

> "*It is important to note that the current DNN models do not capture all aspects of object vision in primates (for example see [23,24]), yet they capture ventral stream processing better than any currently available model type [25].*"

And discussion (lines **468-472**):

> "Regardless, DNNs are mechanistic models that can capture the steps of information processing required to solve visual recognition *of the main object in natural images. While these steps of information processing in DNNs do not capture all aspects of primate vision [23,24],* on a macroscale *they* do map onto successive stages of primate ventral stream processing *[14,15,30]*."

Different models that address certain shortcomings (e.g. recurrence in CORnet, training on Stylized-ImageNet to reduce texture bias) are important steps forward, but they still perform similarly on behavioral and neural benchmarks to more established networks suchs as VGG or AlexNet trained on ImageNet (www.brain-score.org). Validating and comparing all these variants is an active area of research and beyond the scope of the present study.

Thus, we used AlexNet and VGG to compare different behavioral experiments that are generally cited as evidence for higher-level, invariant visual object recognition in rodents. The results show that while some tasks require more stages of DNN processing (up to mid-level conv layers), other tasks surprisingly do not require these deeper representations. We conclude that, taken together, the results suggest that the behavioral studies we evaluate *at best* provide evidence for mid-level processing (in terms of DNN layer depth). The "layer at which the task can be solved" is a principled approach that we use to compare object classification tasks, without requiring the assumption that rats solve the task in the same way. That is not a question we can answer with the current data and model comparisons. We reiterate this general approach in the first paragraph of the introduction (lines **407-411**):

> "We examined the object recognition capabilities of the rodent visual system, by focusing on several studies and formally assessing the level of abstraction required to explain behavioral performance. Using convolutional neural networks, we assessed at which stage of processing each network can solve the task*, to shed light on the extent to which successful generalization performance of rats can be taken as evidence for invariant visual object recognition.*"

Minor comments

- 43: "is not yet proven scientifically. " Please rephrase, empirical insights are not proven.

RE: We changed this to "*tested empirically*".

- Fig 1: "The primate ventral stream transforms visual representations where object manifolds are entangled, into more abstract visual representations where object manifolds are linearly separable." I don't think that's definitely shown yet. I'd phrase more carefully.

RE: We changed this to :

> "The primate ventral stream *is thought to* transform visual representations where object manifolds are entangled, into more abstract visual representations where object manifolds are linearly separable *[12]*."

- 57: You might consider including "S. A. Cadena, G. H. Denfield, E. Y. Walker, L. A. Gatys, A. S. Tolias, M. Bethge, and A. S. Ecker Deep convolutional models improve predictions of macaque V1 responses to natural images PLoS Computational Biology, 2019" in the list of references.

RE: We included this citation.

- 250ff: "As such, this computational approach provides an unprecedented mechanistic understanding of the combined behavioral and neural data available in the literature." I don't understand why your work is a mechanistic understanding.

RE: We changed this to:

> "As such, this computational approach provides a *deeper and more principled* understanding of the combined behavioral and neural data available in the literature."

- The entire paper is about rats. I would reflect that in the title/text and change "rodents" to "rats".

RE: We changed the title to:

> "*Using deep neural network models to evaluate object vision tasks in rats.*"

- Number of trials for behavioral experiments not listed.

RE: We included the numbers of trials in Section 4.1.2 "Data and stimulus extraction" (lines **550-559**, see also our response to the Major comments above):

> "The *behavioral data* were copied from the *values displayed* in Fig. 2B of Zoccolan et al. [1] (*percentage correct based on 70-90 trials for each size and azimuth-rotation and per animal, averaged across* N = 6 rats), and extracted from Figures 1C *and 1D* of Djurdjevic et al. [5] using WebPlotDigitizer 4.2 (https://apps.automeris.io/wpd/; *percentage correct based on an unspecified number of trials for each size of the target object and of three*

distractor objects, as well as for the 30° size of all remaining distractor objects, for each of N =6 rats). For the analyses of the natural video categorization experiment, we used the original movies from Vinken et al. [6], and had access to the original data *which are available at https://osf.io/4w39d/ (percentage correct based on 14-49 [M = 30, SD = 10] trials for each target-distractor combination pooled across* N =5 rats)."

- 423: "always retaining the full set of principle components" I don't understand. You do PCA but keep all PCs? Why not use the original data then?

RE: It is not exactly the same as using the original data. First, by doing this we ensure that the dimensionality of the representation on which the task-classifier is trained is identical for all layers. Second, the principal components were estimated using the stimuli of the training set only and thus retaining only those components will remove variability in the test set that was not present in the training set.

- Fig3d: What does "predicted correct" mean? Correctly predicted rat performance or correctly predicted trials? If the latter how are good and bad performers defined in the DNN?

RE: The label says "predicted % correct", as in the average discrimination performances predicted for good/bad performers (see figure legend). The regression model predicting discrimination performance from DNN features was fit separately for good and poorer performers, which we point out with the following text change (lines **245-249**):

> "We calculated how much of the object and size-level variance in behavioral performance could be explained by the DNN layer activations*, by fitting a linear mapping separately for the data from good and poorer performers* (using the data that was displayed in Figure 1 and 2 of Djurdjevic et al. [5]*; Materials and Methods, Computational modeling, Predicting behavioral performance signatures)*."

- Sec 2.2: Not clear to me what was fitted. Did you train the network to predict the rats' performances or did you train it to solve the task?

RE: The network itself was not trained, but a linear mapping was fit to predict behavioral performance signatures from DNN representations (see section "4.3.4 Predicting behavioral performance signatures"). We made sure to refer to the relevant methods sections in this and other places in the results section to avoid any confusion (see our response to the previous question).

- Sec 2.3: Task not clear to me. What did the rats have to distinguish?

RE: This was a rat versus non-rat two-alternative forced choice task (see also Fig. 4 and "4.1.1 Task paradigms"). We made the following changes to the main results text to make this more clear (lines **265-269**, see also our response to the Major comments above):

> "In this study, rats were trained *on a two-alternative forced choice task* in a visual water maze *(see Materials and Methods, Behavior, Task paradigms)* to classify *five-second* videos featuring a rat (target category), from phase scrambled versions of the target videos and target-matched natural distractor videos featuring various moving objects. *The videos were 24 degrees of visual angle as seen from the choice point where the maze splits in two arms [6].*"

- 127ff: Unclear to me what you predicted. Was it the performance of the rat for each transformation?

RE: For all the behavioral experiments performance is the percentage correct across trials. In this particular case we were talking about the percentage correct across all trials separately for each given stimulus transformation (i.e. combinations of size and azimuth-rotation). We made the following changes to clarify (line **193-201**, see also our response to the Major comments above):

> "*The performance of the rats was not the same for all combinations of size and azimuth-rotation: performance was highest for the combination of the most common viewpoint and size in the training set (i.e. the center object in the purple "cross" in Fig 2a,b), and decreased for objects that deviated from that combination. We call this pattern of performances across object transformations (or – more generally – across images) the behavioral performance signature. To assess whether DNN representations could capture the behavioral results beyond overall generalization accuracy,* we tested whether a linear combination of the activation patterns in a DNN layer could also accurately predict *this* behavioral performance *signature* (Materials and Methods, Computational modeling, Predicting behavioral performance *signatures*)."

- Why use representational dissimilarity and not directly predict the responses. See recent paper by Kornblith et al. (https://arxiv.org/abs/1905.00414) on comparing representations of networks.

RE: Representational dissimilarity analysis is the approach we used to analyse the neural data in the original paper and thus unites both explorations of those data.
