# Supplementary material for: Using deep neural networks to evaluate object vision tasks in rats
Source: PLoS Comput Biol. 2021 Mar 2;17(3):e1008714. doi: 10.1371/journal.pcbi.1008714 (PMC7954349; doi:10.1371/journal.pcbi.1008714)
Supplement: Attachment — Submitted filename: 2nd review.pdf [file pcbi.1008714.s006.pdf]

Summary

Thank you for your detailed responses, the additional experiment, and the revision of the manuscript. I think the manuscript improved a lot. However, I have to re-emphasize one point here which I think it really crucial for the interpretation of your results (I just copy my point and your response here again).

My comment: The training performance of these networks is basically flat everywhere. This means that the task is likely linearly separable on almost all layers. This means that there is likely also a linear function that can solve the test samples perfectly on almost every layer, you just cannot find it from limited training data. I am positive that if you trained on the test trials as well you would find that function. However, if that's the case, the conclusion changes, because the reason for the peak at a certain layer is not representational complexity but your ability to fit the data on limited trials.

Your response: The question is not whether the two classes of stimuli used in the experiments are linearly separable – any set of N randomly labeled, non-collinear points will be linearly separable in ≥N-1 dimensional space, so this point could be made for virtually any behavioral stimulus set given the dimensionality of (early) DNN layers (and visual areas). The critical question is whether a hyperplane optimized for a given training set (i.e. the stimuli used to train rats) will generalize to a new set unseen by the classifier (i.e. the stimuli used to test generalization in rats). Thus our conclusions do not depend on the linear separability of the test set (as this is trivial), but on how well a hyperplane that was optimized for the training set generalizes to the test set.

That being said, we realize “complexity” of representations is not the right phrasing to express the goal of our work here, which is to evaluate task difficulty with DNNs trained on object recognition as a framework to better understand what evidence is contained in previous experiments. For this reason we changed the use of the word complexity in the title and throughout the paper (see also our responses to Reviewers #1 and #2).

I don't agree with your response for the following reason: If you talk about "complexity" (now dropped) or "difficulty", you talk about a property of the task based on the given stimuli and possibly the representations in a layer of a deep network. However, you don't want "difficulty" to depend on the learning algorithm of your linear classifier (e.g. the optimization or regularizer) that you use to predict your targets from a particular layer. In my view, you are confounding two things here which should be untangled in the manuscript: (A) linear separability or (B) how characteristic certain features in a layer are for a problem (I'll make that clearer below).

Re (A): Task difficulty could simply mean whether the problem is linearly separable in a given feature space. You seem to refer to this definition as Fig 1a exactly depicts this situation. In particular, this would mean that (given enough trainings data) there is no linear classifier that can separate the two classes in feature space. However, as I pointed out and you agreed, you cannot detect this case since you don't have

enough data and your task is likely always linearly separable. However, Fig. 1a is misleading in this respect. Your responses to this point are that (a) all behavioral set suffers from this and (b) that it's about the generalization of a hyperplane learned on a training set to a test set. (a) might be true but I don't think it's a valid counter argument against my point, as it just points out a methodological problem. If we adopt your argument (b), then your definition of complexity/difficulty suddenly becomes dependent on the type of linear readout that you use. This is why I suggested to find the linear classifier on the test set as well. Assuming it's a convex problem (which is likely is in most of your cases) being unable to find a separable plane would imply that there is none and you can now adopt (A). However, if there is one for each layer because you have too few datapoints, then you cannot uphold (A) because your measure for difficulty basically becomes flat. I also don't think that your reply is a good counter argument in this case, as your learning algorithm simply fails to find the right hyperplane (maybe it would be better with a different regularizer). In addition, your definition of "difficulty" depends on the actual linear learning algorithm that you used (because there is a separating hyperplane that generalizes well; your algorithm just doesn't find it from the training data). I don't see how this connects to "difficulty" or "complexity" because not having enough data or using the wrong linear classifier shouldn't affect how complex or difficult a task is.

Now you could adopt another interpretation (B): Maybe a representation that is characteristic for a particular task should make that task easy to learn and generalize well. To give you an example for what I mean: Consider a finite degree polynomial on a bounded interval in the real line. You could try to learn that polynomial from a basis of monomials or from a basis of sinusoids. Both should work to arbitrary precision. However, since the original function is a polynomial its basis coefficients are sparse in the monomial basis but not in the Fourier basis. That's why it should be easier (more data efficient) to learn in the monomial basis than in the Fourier basis. In addition, it should extrapolate better. You seem to adopt a similar argument in your paper and response. In this case it's about how well the learned linear function generalizes and how data efficient it can be learned. However, in my opinion this does not really measure difficulty or complexity, but rather how characteristic a particular feature set is for that task (i.e. monomials are characteristic for polynomials but sinusoidals are not, in my example).

Since a lot of your conclusions hinge on the "difficulty" interpretation I ask you to clarify your line of reasoning in this respect. Again, from figure 1A you seem to equate difficulty with linear separability and we agree that you cannot detect this given the limited number of datapoints. Thus, the crucial question is: What do you exactly mean by difficulty and how is that connected to generalization of one particular linear learning algorithm?

Minor comments

- The quality of you plots is really low. I presume it's because PLoS, for some reason, wants/produces tiffs out of what was once a beautiful vector graphic. If that's not the case, please check you graphics. There are a lot of compression artifacts in them.
