# Supplementary material for: Using deep neural networks to evaluate object vision tasks in rats
Source: PLoS Comput Biol. 2021 Mar 2;17(3):e1008714. doi: 10.1371/journal.pcbi.1008714 (PMC7954349; doi:10.1371/journal.pcbi.1008714)

Legend:

- blue: responses to the review comments
- *green italic*: changes in the text that we quote from the manuscript
- **red bold**: line numbers in manuscript file

Reviewer #1

This revision fully addresses the few previous criticisms I had. The change of title removes the potentially-problematic claims about complexity, the inclusion of a randomly-initialised comparison DNN in a Supplementary Analysis is interesting and helps relate this work to other recent explorations of random-network performance in rodents and primates, and the improved detail and clarity in the Methods reporting are welcome. I think this is a strong paper!

RE: We thank the reviewer for her/his positive evaluation.

Reviewer #2

Most issues were addressed.

Minor points

1. Figure 3 and related results: I am still unclear on how the authors divide the DNNs into good and poor performers. Did they train separate DNNs?

RE: We did not divide DNNs into good and poorer performers. Only the rats were split into good and poorer performing rats by the authors of the original paper. We used the *same DNN*, but a *separate linear mapping* to explain the behavioral fingerprints for good and poorer performing rats. We made the following changes to further clarify this (lines **249-263**):

> "*Can the behavioral fingerprints of good and poorer performing rats be explained by a difference in readout from the same DNN representation, or is a higher-level representation required to explain the behavior of good performing rats? To investigate this, we* calculated how much of the object and size-level variance in behavioral performance could be explained*, using* the *same* DNN layer activations *for good and poorer performing rats, but* fitting a *separate* linear mapping for the *two groups as defined before* (using the data that was displayed in Figure 1 and 2 of Djurdjevic et al. [5]; Materials and Methods, Computational modeling, Predicting behavioral performance

signatures). For both good and poorer *performing rats*, the percentage explained variance was with ~85% already near maximum for the first convolutional layer, with an absolute maximum of 91% at pool2 of AlexNet (Fig 3b,c). Surprisingly, no higher-level representation was required to capture the behavioral performance signature of good performers, suggesting that in principle the difference in behavioral performance signature between good and poorer *performing rats* can be explained by a different weighting of features of the same representation *from the same DNN*, in contrast with the idea that good performers relied on more advanced processing of shape information [5].”

2. OSF link does not have any software or data.

RE: The link now contains the code for the behavioral DNN models, as well as the rat behavioral data for the video classification experiment (Fig. 4) and the neural data and RDMs (Fig 5). For the behavioral results of Zoccolan et al. (Fig. 2) and Djurdjevic et al. (Fig. 3) we refer to the original manuscripts, which is where we extracted the data from.

3. Line 72: "... a models ..."

RE: We corrected the typo – thank you for pointing it out.

Reviewer #3

Thank you for your detailed responses, the additional experiment, and the revision of the manuscript. I think the manuscript improved a lot. However, I have to re-emphasize one point here which I think it really crucial for the interpretation of your results (I just copy my point and your response here again).

My comment: The training performance of these networks is basically flat everywhere. This means that the task is likely linearly separable on almost all layers. This means that there is likely also a linear function that can solve the test samples perfectly on almost every layer, you just cannot find it from limited training data. I am positive that if you trained on the test trials as well you would find that function. However, if that's the case, the conclusion changes, because the reason for the peak at a certain layer is not representational complexity but your ability to fit the data on limited trials.

Your response: The question is not whether the two classes of stimuli used in the experiments are linearly separable – any set of N randomly labeled, non-collinear points will be linearly separable in ≥N-1 dimensional space, so this point could be made for virtually any behavioral stimulus set given the dimensionality of (early) DNN layers (and visual areas). The critical question is whether a hyperplane optimized for a given training set (i.e. the stimuli used to train rats) will generalize to a new set unseen by the classifier (i.e. the stimuli used to test generalization in rats). Thus our conclusions do not depend on the linear separability of the test set (as this is trivial), but on how well a hyperplane that was optimized for the training set generalizes to the test set.

That being said, we realize “complexity” of representations is not the right phrasing to express the goal of our work here, which is to evaluate task difficulty with DNNs trained on object recognition as a framework to better understand what evidence is contained in previous experiments. For this reason we changed the use of the word complexity in the title and throughout the paper (see also our responses to Reviewers #1 and #2).

I don't agree with your response for the following reason: If you talk about "complexity" (now dropped) or "difficulty", you talk about a property of the task based on the given stimuli and possibly the representations in a layer of a deep network. However, you don't want "difficulty" to depend on the learning algorithm of your linear classifier (e.g. the optimization or regularizer) that you use to predict your targets from a particular layer. In my view, you are confounding two

things here which should be untangled in the manuscript: (A) linear separability or (B) how characteristic certain features in a layer are for a problem (I'll make that clearer below).

RE: As we explained in our reply quoted above, in the context of the behavioral tasks we are ***not*** talking about difficulty in terms of linear separability of the test set **(A)**, but about "***how well a hyperplane that was optimized for the training set generalizes to the test set***" (akin to your **(B)**). We added the following sentence to the introduction to emphasize the reviewer's distinction early on (lines **105-113**):

> "We evaluated for each DNN layer's representational space whether a linear classifier could generalize from the stimuli used in the training phase of experiments in rats, to the novel stimuli used in the test phase. *Thus, rather than asking whether the stimuli of a given experiment are linearly separable (which is likely true for a sufficiently high dimensional space), we are asking whether a linear classifier easily finds a solution for the training set that generalizes well to the test set. That is, we are asking how well each DNN layer's representational space lends itself to the task the rats were probed with.* We reasoned that if generalization *as observed in rodents* is successful in early DNN layer representations, the task does not require high-level, invariant object representations, and thus one should be careful in interpreting generalization performance of rodents as evidence for invariant object recognition."

It is true that even where our SVM classifier failed to generalize, one can never exclude that there exists a different algorithm that happens to find a solution which does generalize for a particular test set, or vice versa. However, exhaustively exploring the space of all possible classification algorithms in search for that particular classifier would be highly problematic, as we would be merely overfitting to the test set of the experiment in question.

Nevertheless, we want our results to be representative and robust. Just to be sure, we checked this for the dataset with the rat movies for a wide range of algorithms, regularization methods, regularization strengths, and classification models. Apart from a few exceptions of combination of values where the classifier ran into trouble even to learn the classification on the training stimuli, the large majority of combinations result in an outcome (e.g., performance on the test images) that is highly similar to the methods that we used throughout. We have added an overview of these findings as an appendix at the end of this document. Taking it all together, what we show in the paper is representative for what is typically found in a large sample of classifiers.

Re (A): Task difficulty could simply mean whether the problem is linearly separable in a given feature space. You seem to refer to this definition as Fig 1a exactly depicts this situation. In particular, this would mean that (given enough trainings data) there is no linear classifier that can separate the two classes in feature space. However, as I pointed out and you agreed, you cannot detect this case since you don't have enough data and your task is likely always linearly separable. However, Fig. 1a is misleading in this respect. Your responses to this point are that

(a) all behavioral set suffers from this and (b) that it's about the generalization of a hyperplane learned on a training set to a test set. (a) might be true but I don't think it's a valid counter argument against my point, as it just points out a methodological problem. If we adopt your argument (b), then your definition of complexity/difficulty suddenly becomes dependent on the type of linear readout that you use. This is why I suggested to find the linear classifier on the test set as well. Assuming it's a convex problem (which is likely is in most of your cases) being unable to find a separable plane would imply that there is none and you can now adopt (A). However, if there is one for each layer because you have too few datapoints, then you cannot uphold (A) because your measure for difficulty basically becomes flat. I also don't think that your reply is a good counter argument in this case, as your learning algorithm simply fails to find the right hyperplane (maybe it would be better with a different regularizer). In addition, your definition of "difficulty" depends on the actual linear learning algorithm that you used (because there is a separating hyperplane that generalizes well; your algorithm just doesn't find it from the training data). I don't see how this connects to "difficulty" or "complexity" because not having enough data or using the wrong linear classifier shouldn't affect how complex or difficult a task is.

RE: In Fig. 1a we were talking about linear separability of object representations in the ventral stream in general, thus not restricted to a small stimulus set from one of the experiments. To avoid giving the impression that we are assessing linear separability of the experimental stimulus sets we changed the schematic in Fig. 1a to emphasize separability in general and changed the legend accordingly (lines **69-71**):

> “**(a)** The primate ventral stream is thought to transform visual representations where objects are entangled, into more abstract visual representations where objects are *increasingly* separable [12].”

We further scrutinized the text to find for another place where we might (incorrectly) suggest that that we are confounding linear separability of the test set with what we are actually assessing (how easily can a classifier generalize from training to test set?), and we found and corrected one instance in the results section (lines **152-155**):

> “Testing and comparing the performance of these models on behavioral experiments of visual object recognition in rats, provides information about the task difficulty *in terms of* the level of DNN processing steps that would be required to *obtain a representational space that lends itself well to the task*.”

and one in the methods section (lines **641-644**):

> “Each classifier was trained on the experiment’s training set only, and tested for generalization on the test set to assess whether its feature representation can support

the task (i.e., whether the object/category representations *are such that a linear combination of features optimized for the training set generalizes to the test set*)."

As mentioned before, we now also explored a large spectrum of classifiers (see appendix below), showing that the classifiers that we used are very representative for this spectrum. Among this large spectrum, there are maybe two cases that behave like the reviewer suggests, each time with this combination of hyperparameters/algorithms: λ=1000/135, lasso, sparsa (appendix IV). These classifiers are better in generalization to the test set. Interestingly, these are also classifiers that do not manage to do very well on the training set (much lower than most other classifiers). We think it is most prudent to show findings that are representative for what most classifiers do. Importantly, however, the few exception classifiers do not contradict our main conclusion. This conclusion is that representations in low- or mid-level layers are sufficient to simulate rodent behavior. If we were to take the two exception classifiers, then this conclusion would even be stronger as in those cases even the first layer already shows relatively high generalization performance.

Now you could adopt another interpretation (B): Maybe a representation that is characteristic for a particular task should make that task easy to learn and generalize well. To give you an example for what I mean: Consider a finite degree polynomial on a bounded interval in the real line. You could try to learn that polynomial from a basis of monomials or from a basis of sinusoids. Both should work to arbitrary precision. However, since the original function is a polynomial its basis coefficients are sparse in the monomial basis but not in the Fourier basis. That's why it should be easier (more data efficient) to learn in the monomial basis than in the Fourier basis. In addition, it should extrapolate better. You seem to adopt a similar argument in your paper and response. In this case it's about how well the learned linear function generalizes and how data efficient it can be learned. However, in my opinion this does not really measure difficulty or complexity, but rather how characteristic a particular feature set is for that task (i.e. monomials are characteristic for polynomials but sinusoidals are not, in my example).

RE: As you suggest, this is indeed the point we have been making. It measures task difficulty in the sense that a task that involves a more difficult object recognition problem will not be solved by a typical linear classifier without a set of abstract, higher-level features. That is also a good argument to show results that are representative for what a 'typical' classifier does.

Since a lot of your conclusions hinge on the "difficulty" interpretation I ask you to clarify your line of reasoning in this respect. Again, from figure 1A you seem to equate difficulty with linear separability and we agree that you cannot detect this given the limited number of datapoints. Thus, the crucial question is: What do you exactly mean by difficulty and how is that connected to generalization of one particular linear learning algorithm?

RE: To recapitulate, we define task difficulty by the DNN layer rank at which a *typical* linear classifier finds a separating hyperplane for the training set of the task that generalizes well to the test set of the task. For an "easy" task, low-level features lend themselves well to the task,

and stimulus representations in early DNN layers are such that the hyperplane for the training set is easy to learn (which is trivial, but is an important starting point) AND should generalize well to the test set for a typical linear classifier. For a “difficult” task, only higher level features lend themselves well to the task, and thus only stimulus representations in higher DNN layers are such that the hyperplane for the training set generalizes well to the test set.

Given the small sizes of the stimulus sets and the high dimensionality of DNN representations, a linear learning algorithm that generalizes well may exist for each experiment/layer combination. Exhaustively searching for this algorithm for each experiment/layer combination would be circular, and thus to avoid double-dipping we committed a priori to one commonly used learning algorithm (SVM with a standard optimization and hyperparameters). We now also checked that other linear classifiers typically show similar results.

Minor comments

- The quality of you plots is really low. I presume it's because PLoS, for some reason, wants/produces tiffs out of what was once a beautiful vector graphic. If that's not the case, please check you graphics. There are a lot of compression artifacts in them.

RE: This is only in the pdf file generated by PLOS, although each figure page in that pdf has a link saying “Click here to access/download” in the upper right corner to a high res version without artifacts.

APPENDIX I: changing model and regularization strength

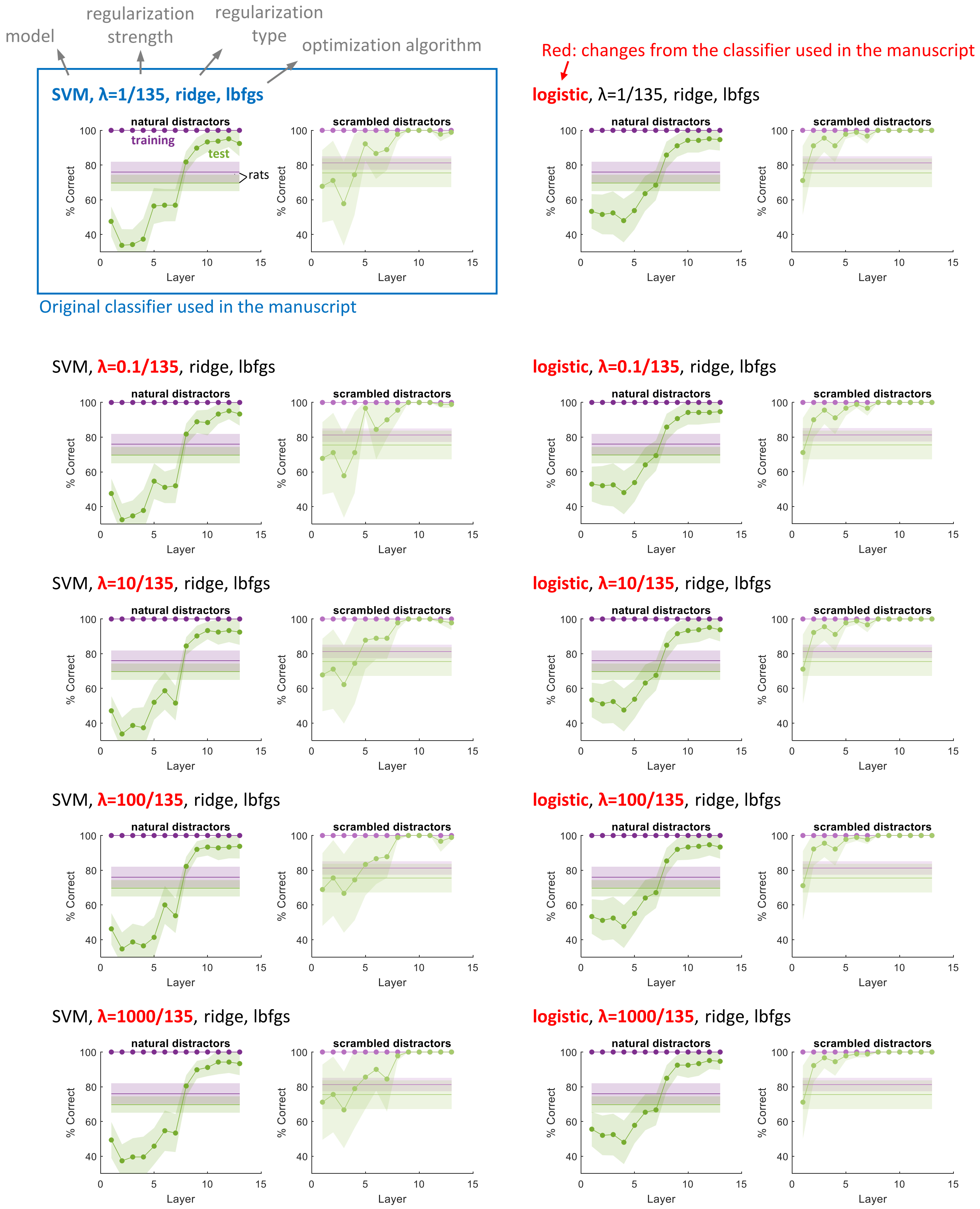

APPENDIX II: changing model and optimization algorithm

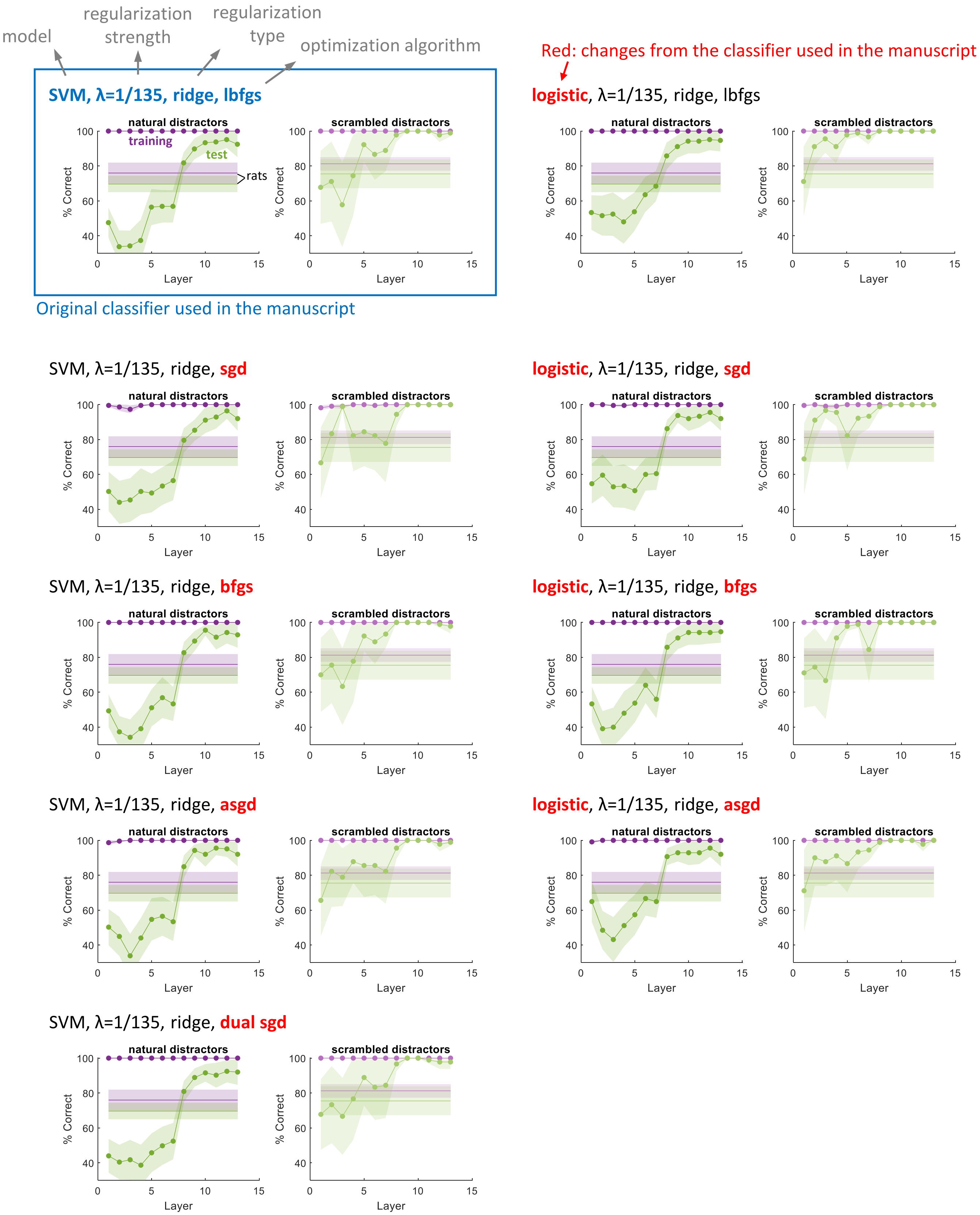

APPENDIX III: changing model, regularization type, and optimization algorithm

model | regularization strength | regularization type | optimization algorithm

Red: changes from the classifier used in the manuscript

SVM, λ=1/135, ridge, lbfgs

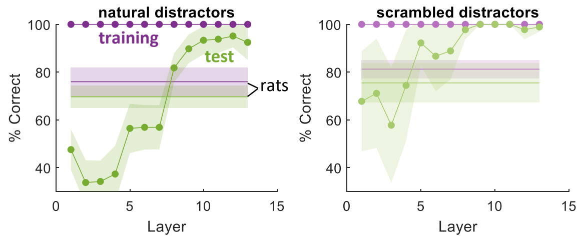

Original classifier used in the manuscript

logistic, λ=1/135, ridge, lbfgs

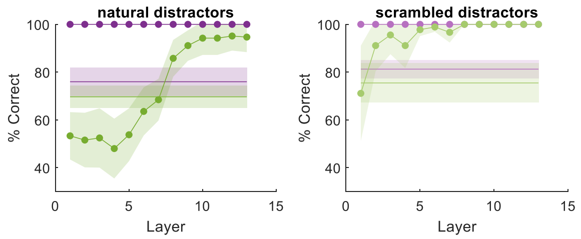

SVM, λ=1/135, **lasso**, **sgd**

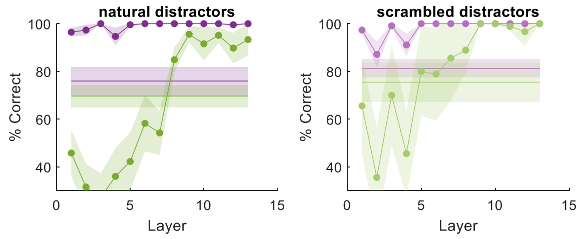

logistic, λ=1/135, **lasso**, **sgd**

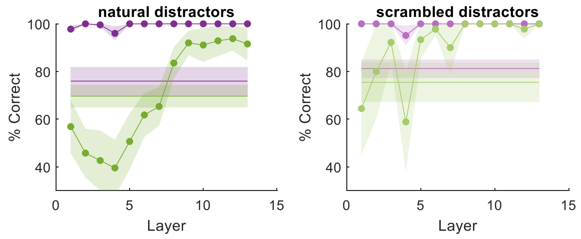

SVM, λ=1/135, **lasso**, **sparsa**

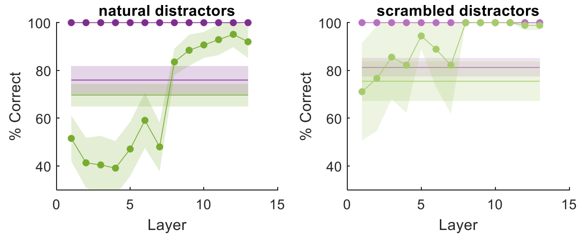

logistic, λ=1/135, **lasso**, **sparsa**

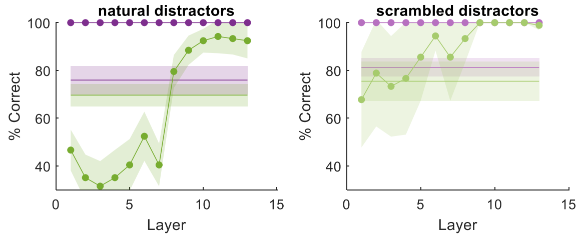

SVM, λ=1/135, **lasso**, **asgd**

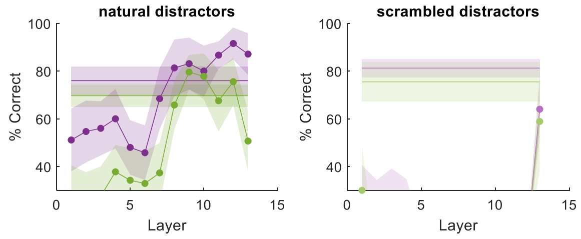

logistic, λ=1/135, **lasso**, **asgd**

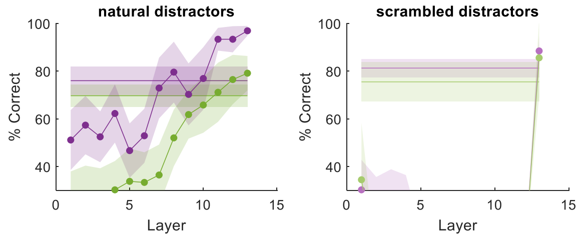

APPENDIX IV: changing model, regularization type, regularization strength, and optimization algorithm

model | regularization strength | regularization type | optimization algorithm

SVM, λ=1/135, ridge, lbfgs

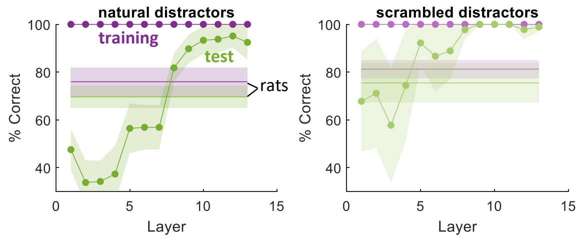

Original classifier used in the manuscript

Red: changes from the classifier used in the manuscript

logistic, λ=1/135, ridge, lbfgs

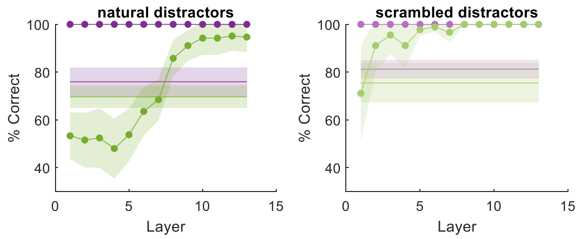

SVM, **λ=0.1/135**, **lasso**, **sparsa**

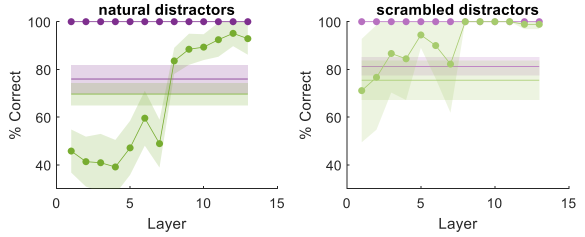

logistic, **λ=0.1/135**, **lasso**, **sparsa**

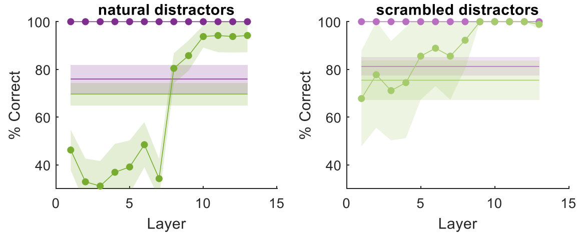

SVM, **λ=10/135**, **lasso**, **sparsa**

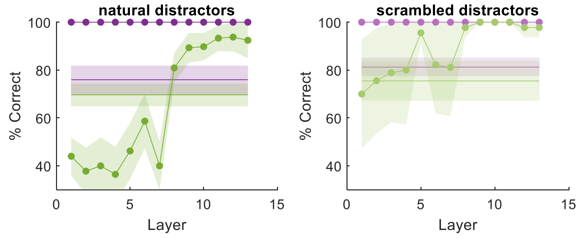

logistic, **λ=10/135**, **lasso**, **sparsa**

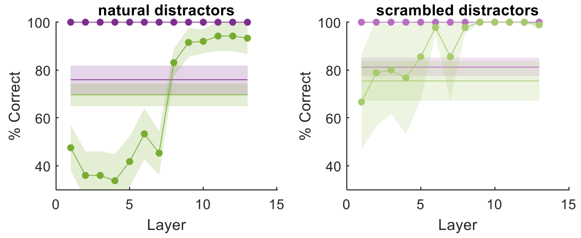

SVM, **λ=100/135**, **lasso**, **sparsa**

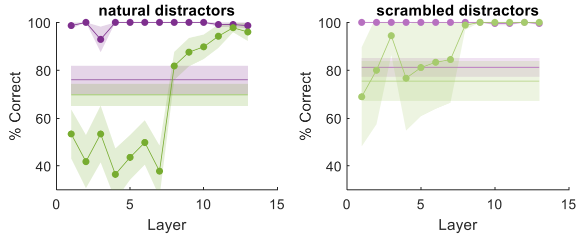

logistic, **λ=100/135**, **lasso**, **sparsa**

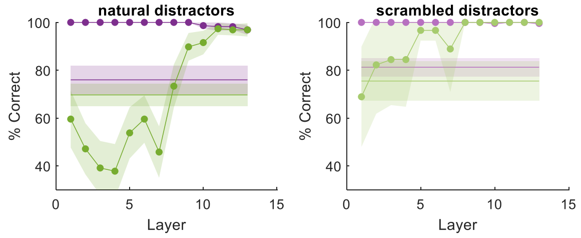

SVM, **λ=1000/135**, **lasso**, **sparsa**

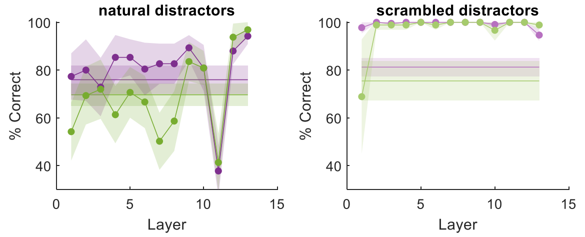

logistic, **λ=1000/135**, **lasso**, **sparsa**

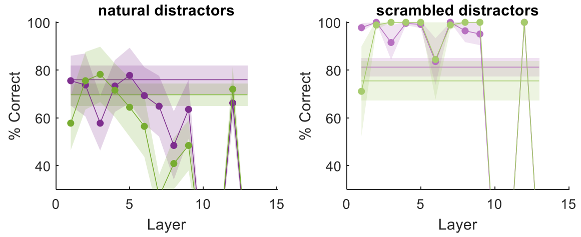

Supplement: Attachment — Submitted filename: response_to_reviews.pdf [file pcbi.1008714.s007.pdf]
